# Influence of Cuttlefish-Ink Extract on Canned Golden Seabream (*Sparus aurata*) Quality

**DOI:** 10.3390/foods13111685

**Published:** 2024-05-27

**Authors:** Beatriz Martínez, Marcos Trigo, Alicia Rodríguez, Santiago P. Aubourg

**Affiliations:** 1 Department of Food Technologies, CIFP Coroso, Avda. da Coruña, 174, 15960 Ribeira, Spain; bmartinezr@edu.xunta.gal; 2 Department of Food Technology, Marine Research Institute (CSIC), c/Eduardo Cabello, 6, 36208 Vigo, Spain; mtrigo@iim.csic.es; 3 Department of Food Science and Chemical Technology, Faculty of Chemical and Pharmaceutical Sciences, University of Chile, c/Carlos Lorca Tobar 964, Santiago 8380494, Chile; arodrigm@uchile.cl

**Keywords:** cuttlefish, ink, golden seabream, canning, lipid hydrolysis, phospholipids, ω3 fatty acids, lipid oxidation, colour, trimethylamine

## Abstract

Four different concentrations of an aqueous extract of cuttlefish (*Sepia* spp.) ink (CI) were introduced, respectively, into the packing medium employed during golden seabream (*Sparus aurata*) canning. The quality parameters of the resulting canned fish were determined and compared to the initial fish and the control canned muscle. An important effect of the CI concentration introduced in the packing medium was proved. The presence in the packing medium of a relatively low CI concentration (CI-2 batch) led to a lower (*p* < 0.05) lipid oxidation development (fluorescent compound formation), lower (*p* < 0.05) changes of colour parameters (L* and a* values), and lower (*p* < 0.05) trimethylamine values in canned fish when compared to control canned samples. Additionally, the two lowest CI concentrations tested led to higher average values of C22:6ω3, ω3/ω6 ratios, and polyene index. On the contrary, the use of the most concentrated CI extract (CI-4 condition) led to a prooxidant effect (higher fluorescence ratio value). In agreement with environmental sustainability and circular economy requirements, the study can be considered the first approach to a novel and valuable use of the current marine byproduct for the quality enhancement of canned fish. On-coming research focused on the optimisation of the CI-extract concentration is envisaged.

## 1. Introduction

As a result of the processing of marine species, seafood industries generate a great quantity of discards [1,2]. Such substrates are known to include a wide variety of healthy and nutritional constituents, but they can also be considered as relevant environmental concerns of coastline areas [3,4]. Among cephalopod discards, ink sacs are generally discarded and thrown without appropriate management. Nevertheless, recent studies carried out on this byproduct have indicated several beneficial properties regarding human health [5,6,7], cosmetic purposes [8], and environmental sustainability [9]. Regarding seafood preservation, previous research has shown relevant antimicrobial [10,11] and antioxidant [12,13,14,15] properties of cephalopod-ink extracts when used in the preparation of seafood substrates.

The preservative behaviour of cephalopod ink has been related to melanin and melanin-free fractions [16]; thus, hydrogen atoms would be donated to any radical included in the reacting medium and would prevent the formation of free-radical formation [17]. The analysis of the melanin-free ink corresponding to splendid squid (*Loligo formosana*) indicated that the highest antioxidant behaviour was present in fractions presenting molecular weights lower than 3 kDa, whose activity was not modified after a heat treatment (30 min at 90 °C) [18]. Based on a chromatographic analysis, Senan [19] proved that the active principle in the methanolic extract obtained from cuttlefish (*Sepia pharaonis*) ink was a peptide molecule, which was shown to be stable during thermal treatment (30 min at 40–100 °C). Different molecular-weight fractions were prepared from water-soluble squid (*Ommastrephes bartrami*) melanin [20]; the authors proved that the highest in vitro antioxidant potency corresponded to fractions corresponding to molecular weights above 10 kDa. Recently, Xie et al. [21] indicated that water-extracted cuttlefish (*Sepia sculenta*) melanin contained a characteristic indole structure with irregular spherical structures and provided a protective effect on kojic and ascorbic acids from light irradiation; this preservative effect was maintained by prolonging the exposure time.

Canning is considered a traditional process that is widely employed and is able to inactivate enzymes and destroy pathogenic and spoilage microorganisms [22,23]. However, the high temperature involved may provoke the destruction of proteins, vitamins, and unsaturated fatty acids (FAs) and lead to a decrease in the nutritional and sensory values [24,25]. Therefore, searching for optimised conditions for the canning process [26] and the use of protective technologies [27] is nowadays considered mandatory. Additional concerns of fish canneries are the availability decrease of traditional species and the need for previous storage of the raw material to be processed [28,29]. In both cases, the use of farmed species can provide a more accurate and convenient raw material in any season of the year.

The current work focused on the employment of cuttlefish (*Sepia* spp.) ink (CI). The basic objective was to study the preservative properties of an aqueous CI extract during the canning process of farmed golden seabream (*Sparus aurata*). This fish species was chosen on the basis of its great production and the interest in introducing farmed species as raw material for canning. In this study, different concentrations of CI were added, respectively, to the packing medium. Quality changes regarding phospholipid content, lipid hydrolysis, FA profile, lipid oxidation, muscle colour and trimethylamine (TMA) value were measured in the initial and canned samples. The effect of the CI concentration employed was investigated.

## 2. Materials and Methods

### 2.1. Analysis of the Initial Ink and Preparation of the Ink Extract

Commercial ink from cuttlefish (*Sepia* spp.) was obtained from Sepink (Vilagarcía de Arousa, Pontevedra, Spain). Once in our laboratory, the ink was subjected to the following analytical determinations: proximate analysis, FA profile, and lipid quality determination.

For it, moisture content was determined as the weight difference before and after 4 h at 105 °C [30]; the results were calculated as g water·kg^−1^ ink. Crude protein content was measured using the Kjeldahl method [30], with a conversion factor of 6.25; the results were calculated as g protein·kg^−1^ ink. Ash content was measured according to the AOAC [30] method; results were calculated as g ash·kg^−1^ ink. Carbohydrates were estimated by difference and expressed as g carbohydrates·kg^−1^ ink.

The lipid content, the FA profile (individual FAs and FA groups and ratios) and the quality (presence of peroxides, thiobarbituric acid reactive substances, and free fatty acids) of the initial ink were determined according to the analytical methods described in Section 2.3 and Section 2.4.

In order to obtain the ink extract, a mixture of CI (40 g) and distilled water (400 mL) was subjected to stirring (30 s), sonication (30 s), and centrifugation (3500× *g* at 4 °C for 30 min) according to previous research [14]. The resulting supernatant was taken. The extraction process was carried out three more times, with all supernatants being pooled together and made up to 1 L with distilled water.

The ink extract was subjected to the proximate analysis according to the methods previously mentioned. Additionally, the total volatile base-nitrogen (TVB-N) content was determined as described previously [31]; results were expressed as mg TVB-N·kg^−1^ ink extract).

### 2.2. Raw Fish and Fish Canning

Fresh farmed golden seabream (*S. aurata*) (44 specimens) (ca. 480–520 g each) were obtained in a local market and carried stored on ice to the laboratory. Then, four individuals were selected and considered as the initial fish. In order to obtain the fillets, the fish individuals were beheaded and eviscerated. After discarding the dark muscle from the fillets, the resulting white muscle was minced and analysed independently in each of the fish individuals (*n* = 4).

On the same day, the remaining fish were divided into four batches (ten individuals per batch) that were considered independently during the current study (*n* = 4). In all the batches, the fillets were obtained after the fish were subjected to beheading and evisceration. Then, 45 g portions of fillets were introduced into small flat cans (105 mm × 60 mm × 25 mm; 150 mL). Two cans were prepared from each individual fish. In each batch, 0, 10, 25, 40, and 80 mL of the above-mentioned CI extract were added, respectively, to the cans as packing media. Cans were filled by the addition of distilled water (95, 85, 70, 55, and 15 mL, respectively). As a result, CTR (canned control), and samples corresponding to CI-1, CI-2, CI-3, and CI-4 packing conditions were prepared, respectively.

The CI concentrations tested in the current work were based on preliminary tests. Thus, the CI-4 condition corresponds to the highest concentration that does not modify the sensory descriptors of canned golden seabream (namely odour and flavour or flesh colour). With the aim of analysing the effect of the CI-extract concentration, three conditions, including lower CI concentrations (i.e., CI-1, CI-2, and CI-3 packing conditions), were also checked.

The cans were vacuum-sealed (SOMME 222, Ezquerra, San Adrián, Navarra, Spain) and sterilised (115 °C, 45 min; *F*_o_ = 7 min) in a steam retort (Presoclave II 75L, JP Selecta, Barcelona, Spain). Once the sterilisation time was finished, the steam was cut off, the remaining steam was flushed away by the use of air, and the cans were cooled by using water at reduced pressure.

After a 3-month storage at room temperature (20 °C), the cans were opened. For it, the liquid part of the can was drained off gravimetrically and filtered by means of a filter paper. Once the dark muscle was discarded, the white muscle of the canned fish was wrapped in filter paper and employed for the different kinds of physico-chemical analyses.

According to the common practice carried out in canneries, a 3-month storage was accomplished. Canning manufacturers recommend that a 2–3-month storage ought to be carried out in order to optimise the sensory acceptability of the commercial canned product [28].

In all cases, chemical reagents and solvents corresponding to the reagent grade were used (Merck, Darmstadt, Germany).

### 2.3. Determination of Lipid Content and Composition in Fish Muscle

The lipid fraction was obtained by extracting the fish’s white muscle according to the Bligh and Dyer [32] method. Quantification was carried out in agreement with Herbes and Allen [33]. Lipid content was calculated as g·kg^−1^ fish muscle.

The free fatty acid (FFA) value was analysed on the lipid extract of the fish muscle in agreement with the Lowry and Tinsley [34] method. Results were calculated as g FFAs·kg^−1^ lipids.

The phospholipid (PL) value was determined by measuring the organic phosphorus in the lipid extract according to the Raheja et al. [35] procedure. Results were calculated as g PLs·kg^−1^ lipids.

Fatty acid methyl esters (FAMEs) were prepared from the lipid extracts by employing acetyl chloride in methanol. The resulting FAMEs were then analysed by gas chromatography (Perkin-Elmer 8700 chromatograph, Madrid, Spain) in agreement with an established procedure [36]. Identification of peaks corresponding to FAMEs was achieved by a comparison of the retention times to those of standard mixtures (Qualmix Fish, Larodan, Malmo, Sweden; Supelco 37 Component FAME Mix, Supelco Inc., Bellefonte, PA, USA). For quantitative purposes, C19:0 was employed as an internal standard. The content of each FA was expressed as g·100 g^−1^ total FAs.

Values regarding FA groups (saturated FAs, STFAs; monounsaturated FAs, MUFAs; polyunsaturated FAs, PUFAs; ω3 and ω6 PUFAs) were calculated by means of values obtained in individual FAs. Additionally, the ω3/ω6 ratio and the polyene index (PI), considered as the C20:5ω3 + C22:6ω3/C16:0 concentration ratio, were calculated.

### 2.4. Assessment of Lipid Oxidation Development in Fish Muscle

The peroxide value (PV) was analysed spectrophotometrically (520 nm) (Beckman Coulter, DU 640; London, UK) on the lipid extract according to the previous research [37]. Results were calculated as meq. active oxygen·kg^−1^ lipids.

The thiobarbituric acid index (TBA-i) was analysed in agreement with Vyncke [38]. Thus, the thiobarbituric acid reactive substances (TBARSs) value was spectrophotometrically measured at 532 nm. Results were calculated as mg malondialdehyde·kg^−1^ muscle.

The formation of interaction compounds produced by the reaction of oxidised lipids and protein-type molecules was determined by fluorescence spectroscopy (Fluorimeter LS 45; Perkin Elmer España; Tres Cantos, Madrid, Spain) in agreement with previous research [39]. The fluorescence ratio (FR) was measured in the aqueous phase obtained from the lipid extraction of the fish muscle.

### 2.5. Determination of Muscle Colour and TMA Value in Fish Muscle

The assessment of colour parameters (L*, a* and b*) was carried out on the fish-muscle surfaces of the raw and canned samples. For it, an instrumental determination (CIE 1976 Lab), performed with a tristimulus Hunter Labscan 2.0/45 colourimeter (Reston, VA, USA), was undertaken. For the analysis of each sample, colour scores were averaged over four determinations, which were taken by rotating the measuring head 90° between triplicate measurements per position.

The TMA-nitrogen (TMA-N) content was analysed using the picrate colourimetric method [40]. Results were calculated as mg TMA-N·kg^−1^ muscle.

### 2.6. Statistical Analysis

As previously indicated, four replicates (*n* = 4) were considered in this work. Data obtained were evaluated by analysis of variance (ANOVA) to explore differences resulting from the effect of the sterilisation and canned storage steps, and the effect of the CI-extract concentration added to the packing medium. For carrying out the comparison of average values, the Tukey HSD procedure was applied. A confidence interval at the 95% level (*p* < 0.05) was taken into account to establish significant differences among batches. The PASW Statistics 18 software for Windows (SPSS Inc., Chicago, IL, USA) was employed.

## 3. Results and Discussion

### 3.1. Composition and Quality of the Initial Ink

The initial ink exhibited the following proximate composition (g·100 kg^−1^): 77.28 ± 0.89 (moisture), 6.52 ± 0.19 (protein), 0.13 ± 0.01 (lipids), 8.93 ± 0.34 (ash), and 7.14 ± 0.45 (carbohydrates). A different composition was reported by Xie et al. [21] for *S. esculenta* ink: 13.9% (proteins), 0.3% (carbohydrates), 0.02% (lipids), and 6.1% (ash).

The FA profile of the initial ink indicated the following composition of individual FAs (g·100 g^−1^ total FAs): 2.45 ± 0.13 (C14:0), 0.69 ± 0.06 (C15:0), 26.68 ± 1.77 (C16:0), 1.38 ± 0.12 (C16:1ω7), 2.63 ± 0.09 (C17:0), 18.52 ± 1.29 (C18:0), 8.34 ± 0.33 (C18:1ω9), 1.91 ± 0.11 (C18:1ω7), 4.01 ± 0.16 (C18:2ω6), 2.99 ± 0.19 (C20:1ω9), 0.38 ± 0.01 (C20:2ω6), 6.36 ± 0.34 (C20:4ω6), 0.42 ± 0.01 (C22:1ω9), 6.17 ± 0.53 (C20:5ω3), 1.55 ± 0.12 (C22:4ω6), 0.56 ± 0.03 (C24:1ω9), 1.54 ± 0.13 (C22:5ω3), and 13.27 ± 1.35 (C22:6ω3). Therefore, the most abundant FA was C16:0, followed by C18:0 and C22:6ω3; other relatively abundant FAs were C18:1ω9, C20:4ω6, and C20:5ω3.

The content of the FA groups was also determined (g·100 g^−1^ total FAs). According to the individual FA profile, STFAs were shown to be the most abundant group (51.03 ± 3.31), while MUFAs depicted the lowest levels (15.62 ± 0.75). The values obtained for PUFAs and ω3 PUFAs were 33.35 ± 2.63 and 21.05 ± 2.01, respectively. Additionally, the following FA ratio values were obtained: 1.71 ± 0.08 (ω3/ω6 ratio) and 0.74 ± 0.12 (PI).

Regarding the lipid quality of the initial ink, the following lipid oxidation values were observed: 0.81 ± 0.11 (PV) and 0.07 ± 0.01 (TBA-i). According to general research on seafood quality, these values correspond to a low-rancidity substrate susceptible to being employed in further activities [39,41]. A relatively high FFA value was detected (i.e., 327.13 ± 6.21 g·kg^−1^ lipids); this high value can be explained on the basis of the low total lipid content and the inverse ratio reported between total lipid and FFA values [42].

The ink extract showed moisture and ash contents of 996.04 ± 0.12 and 0.01 ± 0.00 g·kg^−1^, respectively; no presence of lipids and proteins was detected. The determination of the TVB-N content indicated a 13.1 ± 0.4 mg·kg^−1^ score.

### 3.2. Determination of Total Lipid, FFA, and PL Values in Fish Muscle

Values obtained for total lipid content in fish muscle are depicted in Table 1. Canning and canned storage led to a general lipid content increase that can be explained on the basis of moisture loss from the muscle during the heating treatment [29,36]. No differences (*p* > 0.05) among the canned samples were obtained, all values being included in the 20.79–24.03 g·kg^−1^ muscle.

A comparison between the initial fish and canned fish corresponding to the control condition indicated a marked increase (*p* < 0.05) in the FFA value as a result of the sterilisation and canned storage steps (Table 1). All CI-treated fish showed higher mean values of FFAs than the control canned fish. However, the differences were not found significant (*p* > 0.05).

In the present work, the FFA value can be influenced by different factors. First, the sterilisation process would provoke the hydrolysis of higher-molecular-weight lipid classes such as triacylglycerols (TAGs) and PLs [24,43]. Then, FFAs can be oxidised or broken-down during the sterilisation process, providing a greater accessibility to oxygen and other pro-oxidant molecules when compared to the TAG and PL classes [44,45]; consequently, this effect would lead to a decrease in the FFA content. The current results have shown that the first effect (i.e., thermal hydrolysis of TAGs and PLs) was more important than the second one (i.e., thermal breakdown of FFAs). All CI-treated fish showed higher average FFA levels than the canned control condition; however, the differences were not found significant (*p* > 0.05).

According to the present study, a preservative effect on FFAs was inferred by Barbosa et al. [36] during Atlantic Chub mackerel (*Scomber colias*) canning by the presence of an aqueous extract of *F. spiralis* in the packing medium. The same authors [31] proved a higher FFA retention in canned Atlantic mackerel (*Scomber scombrus*) by the presence of *Bifurcaria bifurcata* extracts in the packing medium. On the contrary, a lower FFA content was detected in canned Chub mackerel (*S. colias*) by including an aqueous extract of octopus (*Octopus vulgaris*) cooking liquor [46]. In the meantime, no effect was proven in canned Chub mackerel (*S. colias*) by the addition of *Ulva lactuca* extracts [36].

Regarding the PL fraction in the current study, the content revealed a marked decrease (*p* < 0.05) as a result of the sterilisation treatment and canned storage (comparison between the initial fish and control canned fish) (Table 1). However, all CI-treated fish showed higher average values than the control canned condition. Remarkably, canned muscle corresponding to the CI-1 condition did not show a significant loss (*p* > 0.05) of the PL content when compared to the initial fish.

Great attention has been accorded to PLs according to their high bioavailability and preserving effect as delivery systems for different kinds of diseases [47,48]. Based on the needs and requirements expressed by the food and pharmaceutical industries, great efforts are being made toward the retention of the PL fraction in seafood as it presents high PUFA levels [49,50].

Previous studies regarding the effect of antioxidant compounds included in the packing system on the PL content in canned fish are scarce. The addition of an aqueous extract of *Fucus spiralis* to the packing medium of brine-canned Chub mackerel (*S. colias*) led to a preserving effect on the PL composition [36]. Similarly, Méndez et al. [51] proved that there was a retention of the PL content in brine-canned horse mackerel (*Trachurus trachurus*) by an addition to the packing medium of an aqueous extract of octopus (*O. vulgaris*) cooking liquor.

### 3.3. Analysis of the FA Profile of Fish Muscle

The individual FA composition of the initial and canned fish is shown in Table 2. In all cases, C18:2ω6 (linoleic acid) (26.88–28.27% range) and C18:1ω9 (oleic acid) (23.67–26.77% range) were the most abundant FAs, followed by C16:0 (palmitic acid) (15.02–16.53% range) and C22:6ω3 (docosahexaenoic acid, DHA) (8.62–11.94% range).

According to the nutritional and healthy properties of seafood in general, quality changes related to the FA values in the present study will be addressed to the ω3 PUFA fraction (eicosapentaenoic acid, EPA; DHA; docosapentaenoic acid, DPA; total ω3 PUFAs) and the FA ratios (ω3/ω6 and PI).

A comparison of the samples corresponding to the initial fish and the canned control condition revealed a loss (*p* < 0.05) of EPA and DHA values (Table 2) as a result of the sterilisation and the canned storage steps. Regarding the CI treatment, all CI-treated fish showed higher average EPA values than the canned control; a significant loss could not be inferred (*p* > 0.05) in treated samples when compared to the initial fish. For the DHA value, the samples corresponding to the CI-1 and CI-2 conditions showed higher average values than the canned control fish. On the contrary, samples corresponding to CI-3 and CI-4 conditions revealed lower average values. Regarding the DPA values, a remarkable loss (*p* < 0.05) was detected after the sterilisation and canned storage steps (Table 2). The highest DPA average value was detected in the canned fish corresponding to the CI-2 condition and did not provide differences with the initial fish.

The total ω3 PUFA content showed a similar profile to the DHA value (Table 3). Thus, all canned samples (control and CI treated) underwent a content decrease (*p* < 0.05) in comparison to the initial fish, and no significant effect (*p* > 0.05) was concluded as a result of the CI addition to the packing medium. Notably, the samples corresponding to CI-1 and CI-2 conditions showed higher average values of total ω3 FAs than the counterpart canned samples.

The analysis of the ω3/ω6 ratio indicated a marked decrease (*p* < 0.05) as a result of the sterilisation and canned storage steps (Table 3). Therefore, it could be inferred that ω3 FAs have been more susceptible to heat treatment than those belonging to the ω6 series. A comparison among the canned samples (CI treated and control) did not show a significant effect (*p* > 0.05) of the presence of the CI extract in the packing medium. However, and in agreement with current results on the DHA content (Table 3), the CI-1 and CI-2 conditions led to the highest average values of the ω3/ω6 ratio.

Table 3 also includes values of the STFA, MUFA, and PUFA groups in the initial and canned fish samples. In all cases, the PUFA group was shown to be the most abundant (*p* < 0.05). On the contrary, the STFA group provided the lowest (*p* < 0.05) levels in all the kinds of fish samples. As a result of the sterilisation step and canned storage, a decrease (*p* < 0.05) of the PUFA content was detected, but no change (*p* > 0.05) could be inferred for the STFA and MUFA groups. Regarding the CI effect, no differences (*p* > 0.05) could be observed between the canned control and the CI-treated samples for any of the FA groups.

The PI is considered a valuable and practical tool for measuring the possible loss of the PUFA value during marine-species canning or processing in general [36,41,51]. In the present study, this quality parameter showed an average decrease as a result of the thermal treatment and the canned storage (Table 3). Notably, canned fish corresponding to the CI-1 condition did not reflect a significant loss (*p* > 0.05) when compared to the initial fish. Compared to the control canned condition, all CI-treated samples provided higher than average values for this index; however, the differences were not found to be significant (*p* > 0.05). No previous research accounts for the effect of cuttlefish ink on PUFA compounds in canned fish. However, a preservative effect of the cuttlefish (*Sepia officinalis*) ink on PUFA compounds (i.e., higher PI) was already proven by Trigo et al. [14] during a heating treatment of the fish in a model system. The addition to the packing medium of preservative compounds obtained from marine sources has shown a preservative effect on the PUFA content. Thus, Ortiz et al. [52] showed a PI retention of canned Atlantic salmon (*Salmo salar*) muscle when packed in a medium including an ulte (basal part of alga *Durvillaea antarctica*) extract; however, no differences were obtained in such a study when other algae (cochayuyo, frond of *D. antarctica*; *U. lactuca*; and *Pyropia columbina*) extracts were included in the packing system. Higher PI scores were observed in canned Atlantic mackerel (*S. scombrus*) by the addition of *B. bifurcata* extracts [31] and in canned Chub mackerel (*S. colias*) by the addition of *F. spiralis* or *U. lactuca* extracts [36]. Recently, the addition of an aqueous extract of octopus (*O. vulgaris*) cooking liquor to the packing medium led to a higher PI in canned Chub mackerel (*S. colias*) [46].

### 3.4. Determination of Lipid Oxidation in Fish Muscle

The presence of peroxides (primary oxidation compounds) was shown to be low (i.e., < 2.07) in all the samples included in the current study (Table 4). In most cases, differences among the samples were not significant (*p* > 0.05). However, a higher (*p* < 0.05) peroxide content in canned samples corresponding to the CI-1 condition than in the canned control was observed. No effect (*p* > 0.05) of the sterilisation and canned storage steps could be inferred.

As in the case of the peroxide determination, the levels detected for the TBARSs were low, with all values being below the 0.14 score (Table 4). No effect (*p* > 0.05) of the presence of the CI extract in the packing medium could be outlined on the TBARS formation. No effect (*p* > 0.05) was also inferred as a result of the sterilisation and canned storage steps.

**Figure 1:**
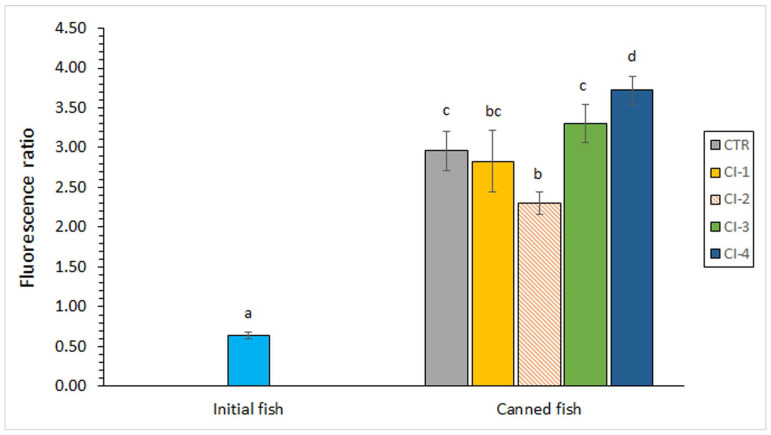
Fluorescence ratio in initial and canned fish, including different concentrations of cuttlefish ink (CI) in the packing medium. Mean values of four replicates (*n* = 4); standard deviations are indicated by bars. Different letters (a, b, c, d) denote significant differences (*p* < 0.05). Abbreviations of sample names as expressed in Table 1.

Interaction compound formation is depicted in Figure 1. A comparison between the initial fish and fish corresponding to the canned control condition showed a remarkable (*p* < 0.05) increase as a result of the canning process and canned storage. Among canned samples, the lowest average values were obtained in fish corresponding to the CI-1 and CI-2 packing conditions. An inhibitory effect (*p* < 0.05) on the fluorescent compound formation was proven by employing the CI-2 packing condition when compared to the canned control samples. On the contrary, the CI-4 condition led to an increased formation (*p* < 0.05) of such kinds of compounds. This dependency on the CI concentration employed is in agreement with the results previously shown regarding the DHA (Table 2) and ω3/ω6 ratio (Table 3) values.

During seafood thermal processing in general, the levels detected for the different lipid oxidation compounds can be considered the result of heating formation and also of heating breakdown [24,25]. According to the low presence of primary and secondary lipid oxidation compounds in the present canned samples, no effect of the CI extract could be inferred on the content of such deteriorative compounds. However, an inhibitory effect on the formation of fluorescent compounds was observed if the CI-2 condition is considered. On the contrary, a prooxidant effect was obtained if the CI-4 condition is taken into account. Therefore, a selective effect of the CI concentration added to the packing medium could be concluded.

An explanation for this contradictory effect found in the CI-2 and CI-4 batches is difficult to give, since an analysis of the molecules responsible and included in the CI extract was not carried out in the current study. However, previous research has proven that the antioxidant activity of an antioxidant compound may depend on different kinds of factors, such as the lipid composition, antioxidant concentration, temperature, oxygen pressure, and the presence of other antioxidants and food constituents [53,54]. Closely related to the present research, Agustini et al. [13] subjected salted-boiled milkfish to two different concentrations (0.75 and 1.50%) of melanin-free ink extract; a prolonged shelf-life time of up to 9 days was obtained with the less-concentrated treatment. Regarding concrete antioxidant compounds, α-tocopherol may behave as a prooxidant at higher concentrations, when prooxidants such as transition metals are present [55]. In the presence of added iron, the effectiveness of different natural antioxidants was reduced and, in some cases, resulted in a prooxidant effect [56]. During a linoleic acid-model system, α-tocopherol showed both antioxidant and prooxidant effects depending on the concentration and the simultaneous presence of Cu (II) and ascorbate [57]. A remarkable antioxidant effect was proven for ascorbate in steam- and microwave-cooked fish [58]; however, above critical values and in agreement with the present research, ascorbate showed pro-oxidant properties. Additionally, Medina et al. [59] proved a remarkable effect of concentration on the antioxidant capacity of polyphenol compounds during the thermal treatment of tuna (*Thunnus alalunga*).

No previous results are available, to the best of our knowledge, regarding the effect on the canned seafood quality of ink extracts obtained from cephalopod species. However, an aqueous extract of cuttlefish (*S. officinalis*) ink led to an inhibitory effect on the formation of conjugated dienes and trienes and fluorescent compounds during a heating treatment of fish in a model system [14]. Additionally, a preservative effect of cephalopod ink has already been proven on non-thermally treated seafood. Thus, the addition of melanin-free ink from splendid squid (*L. formosana*) led to a remarkable decrease in primary and secondary lipid oxidation development in chilled (15 days in ice) mackerel (*Rastrelliger kanagurta*) muscle [18]. Vate et al. [12] indicated a reduction of the peroxide and TBARS formation in refrigerated (20 days at 4 °C) surimi gel sardine (*Sardinella albella*) by the addition of melanin-free ink from splendid squid (*L. formosana*); additionally, a content reduction of nonanal and 2-decenal was reported. Tilapia (*Sparus latus*) fillets were treated with different dosages of melanin-free extract obtained from *S. esculenta* ink [60]; the authors detected an inhibitory effect on TBARS formation in fish fillets during the refrigerated storage (4 °C). Agustini et al. [13] observed an increased shelf-life time of soft-boned milkfish by previous spraying with squid-ink solutions; an antioxidant effect was inferred during storage at 10 °C, 20 °C and 30 °C of fish. An inhibitory effect on peroxide content was detected by Essid et al. [61] in smoked sardines (*Sardinella aurita*) during cold storage (35 days at 4 °C) by previous soaking in a cuttlefish (*S. officinalis*)-ink solution; furthermore, an extension of the shelf-life time was observed.

The presence of macroalgae extracts in the packing medium of canned fish has shown preservative effects. Thus, a decrease in the TBARS level was observed in canned Atlantic salmon (*S. salar*) by the addition of algae (*D. antarctica, U. lactuca,* or *P. columbina*) extracts [52] and a decrease in the fluorescent compound formation was proven in canned Atlantic mackerel (*S. scombrus*) by the presence of *B. bifurcata* extracts [31] and in canned Chub mackerel (*S. colias*) by including algae (*F. spiralis* or *U. lactuca*) aqueous extracts [36]. Regarding the employment of waste substrates produced during seafood processing, a lower fluorescent compound formation was detected in canned horse mackerel (*T. trachurus*) by including an aqueous extract of octopus (*O. vulgaris*) cooking liquor in the packing medium [51].

### 3.5. Assessment of Colour Changes in Fish Muscle

A remarkable increase (*p* < 0.05) of the L* value was detected as a result of the thermal treatment and canned storage (Table 5). This increase was partially avoided in all cases by the presence of the CI extract in the packing medium. Thus, all CI-treated fish revealed lower (*p* < 0.05) L* values than the control canned fish. Additionally, canned fish corresponding to the CI-1 condition did not provide differences (*p* > 0.05) with the initial fish.

An average decrease in the a* value was observed by comparing the initial fish and all kinds of the canned samples (Table 5). This decrease was significant (*p* < 0.05) in the case of the canned control samples but not (*p* > 0.05) in the CI-treated canned fish. No effect (*p* > 0.05) of the CI concentration added to the packing medium could be inferred from this colour parameter.

A comparison between the initial fish and all the kinds of canned samples indicated a relevant increase of the average b* value by means of the sterilisation process and the canned storage (Table 5). The addition of the CI extract to the packing medium led to lower mean b* values than the canned control fish; however, the differences were not found to be significant (*p* > 0.05). The lowest average value was observed for canned fish corresponding to the CI-1 condition.

In agreement with the great influence on the appearance and acceptability of all kinds of seafood, the determination of changes in muscle colour during processing has attracted great attention [62]. The current evolution of colour parameters during the canning process is in agreement with previous studies regarding the heating treatment of seafood. In general, the canning process has led to increased L* and b* values and to a* value decreases [63,64,65].

No previous research has focused on the influence of cephalopod ink on colour changes in canned seafood. However, the presence in the packing medium of preservative compounds has proven valuable effects. Thus, the addition of an aqueous extract of *B. bifurcata* to the packing medium of canned Atlantic mackerel (*S. scombrus*) led to decreased L* and b* values [31]. Recently, Méndez et al. [51] obtained lower L* and b* values in canned horse mackerel (*T. trachurus*) by the addition of an aqueous extract of octopus (*O. vulgaris*) cooking liquor to the packing medium.

### 3.6. Determination of TMA Content in Fish Muscle

**Figure 2:**
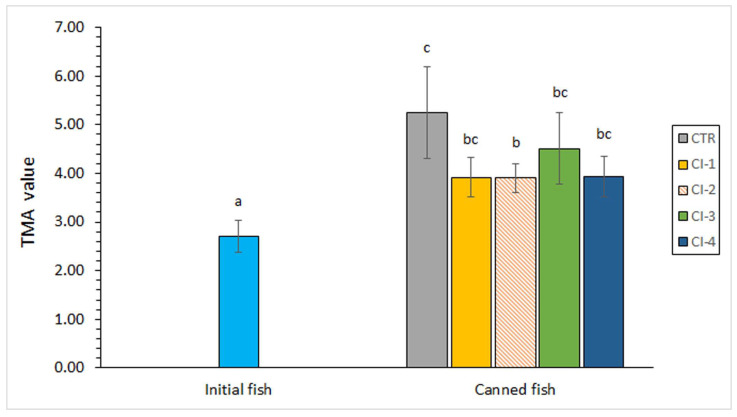
Trimethylamine (TMA) value in initial and canned fish, including different concentrations of cuttlefish ink (CI) in the packing medium. Average values of four replicates (*n* = 4); standard deviations are indicated by bars. Different letters (a, b, c) denote significant differences (*p* < 0.05). Abbreviations of sample names as expressed in Table 1.

A relevant (*p* < 0.05) TMA content increase was observed in all canned samples when compared to the initial fish (Figure 2). Lower average values were found in CI-treated fish when compared to the counterpart canned control. The lowest mean values were observed in the fish samples corresponding to the CI-2 condition, which were significantly lower (*p* < 0.05) than those corresponding to the canned control samples. As for the lipid quality retention (FR, Figure 1), a selective effect of the CI concentration could be inferred.

TMA is recognised as a remarkable deteriorative compound whose value can reflect the quality degradation of seafood subjected to different kinds of technological procedures [66]. Unfortunately, no previous studies are available, to the best of our knowledge, regarding the effect of cephalopod-ink extracts on the TMA value of canned seafood. However, an inhibitory effect has already been proven in non-thermally treated seafood. Thus, Karim et al. [11] demonstrated that a previous squid melanin-free ink soaking from the squid (*Loligo duvaucelli*) led to an increased shelf-life time and a decrease of the TMA and TVB values during refrigerated (4 °C for 15 days) storage. Sadok et al. [67] proved that a previous cuttlefish (*S. officinalis*)-ink soaking led to an inhibitory effect on the formation of off-odour compounds (TMA and TVB values) in cold-stored (0 °C for 12 days and −2 °C for 23 days) peeled shrimp (*Penaeus kerathurus*). Shi et al. [10] subjected yellowfin seabream (*S. latus*) to a marinated treatment with a melanin-free extract from squid ink; as a result, an inhibitory effect on TVB formation was detected in the fish during refrigerated storage at 4 °C. Tilapia (*S. latus*) fillets were subjected to melanin-free extracts obtained from *S. esculenta* ink [60]; the authors observed an inhibitory effect on TVB formation in fish fillets during refrigerated storage (4 °C). Recently, an inhibitory effect on the formation of TVB and TMA was detected by Essid et al. [61] in smoked sardines (*S. aurita*) during cold storage (35 days at 4 °C) by previous soaking in a cuttlefish (*S. officinalis*)-ink solution.

Previous research has also addressed the effect resulting from the addition to the packing medium of preservative compounds obtained from marine sources on the TMA value of canned seafood. As in the present results, an inhibitory effect on TMA formation was detected in canned Chub mackerel (*S. colias*) by the addition of an aqueous *F. spiralis* extract to the packing medium [68]. On the contrary, no effect on the TVB and TMA values was detected in Atlantic mackerel (*S. scombrus*) [31] and Chub mackerel (*S. colias*) [36] by the presence of aqueous extracts of several macroalgae.

## 4. Conclusions

The present research provides a first approach for the employment of a CI extract for the quality enhancement of canned fish. However, the degree of this effect was shown to be notably influenced by the concentration employed. Thus, the employment of the CI concentration corresponding to the CI-2 condition led to a lower lipid oxidation development (fluorescent compound formation), lower colour changes (L* and a* parameters) and a lower TMA value in canned fish when compared to the control canned samples. Although differences were not significant, higher average values of DHA, ω3/ω6 ratio, and PI were detected in canned fish corresponding to the CI-1 and CI-2 conditions. On the contrary, the use of the most concentrated CI extract (CI-4 condition) led to a prooxidant effect (higher FR value) when compared to the control canned fish.

On the basis of the notable effect of the CI concentration employed, further research that takes into account an optimisation design (i.e., response surface methodology) is considered necessary in order to employ the most convenient CI concentration for the canning processing of the current farmed species. Additionally, further research is envisaged to carry out the analysis of the CI extract and, therefore, the molecules involved in the preservative properties detected in the present study. This research is in agreement with the current requirements for the search for new and natural sources of preservative compounds and also agrees with general commitments for environmental sustainability and circular economy.

## Figures and Tables

**Table 1:** Total lipid (TL), free fatty acid (FFA), and phospholipid (PL) values * in initial and canned fish, including different concentrations of cuttlefish ink (CI) in the packing medium **.

Initial or Canned Sample	Lipid Determination		
	TL (g·kg^−1^ Muscle)	FFA (g·kg^−1^ Lipids)	PL (g·kg^−1^ Lipids)
Initial fish	13.62 a (1.64)	0.49 a (0.05)	39.69 b (3.84)
CTR	21.15 b (2.71)	1.58 b (0.31)	29.30 a (3.74)
CI-1	20.79 b (1.76)	1.90 b (0.26)	35.01 ab (3.39)
CI-2	24.03 b (3.09)	1.87 b (0.19)	27.01 a (4.51)
CI-3	22.19 b (3.60)	1.96 b (0.60)	28.75 a (3.46)
CI-4	21.47 b (2.82)	1.97 b (0.27)	29.42 a (3.14)

* Mean values of four replicates (*n* = 4); standard deviations are indicated in brackets. In each column, different letters (a, b) denote significant differences (*p* < 0.05). ** Abbreviations: CTR (canned control), CI-1, CI-2, CI-3, and CI-4 (canned samples including increasing concentrations of the CI extract in the packing medium, according to Section 2).

**Table 2:** Fatty acid (FA) composition * of initial and canned fish, including different concentrations of cuttlefish ink (CI) in the packing medium **.

FA	Initial Fish	CTR	CI-1	CI-2	CI-3	CI-4
14:0	1.33 a (0.07)	1.84 c (0.02)	1.67 b (0.04)	1.85 c (0.11)	1.74 bc (0.11)	1.73 c (0.20)
15:0	0.25 a (0.02)	0.25 a (0.01)	0.28 a (0.02)	0.28 a (0.03)	0.26 a (0.02)	0.26 a (0.02)
16:0	16.53 c (0.45)	15.80 ab (0.31)	15.76 b (0.13)	15.58 ab (0.46)	15.02 ab (0.52)	15.02 a (0.46)
16:1ω7	2.30 a (0.06)	2.67 b (0.18)	2.63 b (0.19)	3.00 bc (0.35)	3.20 c (0.31)	2.96 bc (0.23)
17:0	0.35 a (0.02)	0.37 a (0.02)	0.37 a (0.02)	0.38 a (0.03)	0.36 a (0.02)	0.37 a (0.02)
18:0	4.72 a (0.25)	4.63 a (0.24)	4.71 a (0.15)	4.91 a (0.19)	4.76 a (0.17)	4.89 a (0.22)
18:1ω9	23.67 a (1.18)	25.37 ab (1.04)	25.58 ab (0.94)	25.69 ab (1.14)	26.77 b (1.03)	26.00 b (1.01)
18:1ω7	3.10 a (0.26)	3.44 a (0.35)	3.20 a (0.12)	3.21 a (0.14)	3.35 a (0.13)	3.25 a (0.13)
18:2ω6	26.88 a (0.31)	28.17 c (0.44)	27.57 bc (0.39)	26.67 ab (0.94)	27.73 bc (0.71)	28.27 bc (0.79)
20:1ω9	0.95 a (0.07)	1.34 a (0.12)	1.20 b (0.04)	1.38 b (0.14)	1.28 b (0.11)	1.24 b (0.10)
20:2ω6	1.00 a (0.07)	1.04 c (0.08)	0.97 a (0.07)	1.11 a (0.08)	0.94 a (0.10)	0.96 a (0.12)
20:4ω6	1.20 a (0.09)	1.10 b (0.10)	1.13 a (0.08)	1.14 a (0.07)	1.01 a (0.09)	1.08 a (0.10)
22:1ω9	0.28 a (0.03)	0.31 a (0.04)	0.28 a (0.01)	0.34 a (0.05)	0.29 a (0.02)	0.27 a (0.03)
20:5ω3	2.26 b (0.12)	1.99 a (0.10)	2.06 ab (0.20)	2.10 ab (0.16)	2.00 ab (0.12)	2.06 ab (0.24)
22:4ω6	0.27 a (0.02)	0.25 a (0.01)	0.24 a (0.01)	0.28 a (0.01)	0.26 a (0.02)	0.26 a (0.04)
24:1ω9	0.50 a (0.04)	0.48 a (0.03)	0.49 a (0.02)	0.51 a (0.04)	0.47 a (0.05)	0.48 a (0.05)
22:5ω3	2.39 b (0.08)	2.15 a (0.10)	2.12 a (0.12)	2.36 ab (0.12)	2.09 a (0.07)	2.16 ab (0.20)
22:6ω3	11.94 b (0.74)	8.68 a (1.14)	9.66 a (0.95)	9.11 a (0.34)	8.35 a (1.13)	8.62 a (0.95)

* Mean values of four replicates (*n* = 4); standard deviations are indicated in brackets. In each row, different letters (a, b, c) denote significant differences (*p* < 0.05). ** Abbreviations of sample names as expressed in Table 1.

**Table 3:** Fatty acid (FA) group and ratio values * of initial and canned fish, including different concentrations of cuttlefish ink (CI) in the packing medium **.

	Initial Fish	CTR	CI-1	CI-2	CI-3	CI-4
ω3 PUFAs	16.62 b (0.94)	12.88 a (1.26)	13.89 a (1.23)	13.58 a (0.19)	12.50 a (1.15)	12.90 a (1.30)
ω3/ω6	0.57 b (0.03)	0.42 a (0.05)	0.46 ab (0.04)	0.47 a (0.01)	0.42 a (0.05)	0.42 a (0.05)
STFAs	23.20 a (0.64)	22.91 a (0.56)	22.78 a (0.16)	23.02 a (0.61)	22.16 a (0.61)	22.29 a (0.52)
MUFAs	30.82 a (1.56)	33.63 ab (1.48)	33.41 ab (1.16)	34.18 ab (1.69)	35.40 b (1.43)	34.23 b (1.10)
PUFAs	45.98 b (0.93)	43.34 a (1.01)	43.81 ab (1.20)	42.79 a (1.09)	42.45 a (0.85)	43.48 a (0.71)
PI	0.86 b (0.03)	0.68 a (0.06)	0.75 ab (0.07)	0.72 a (0.01)	0.69 a (0.05)	0.71 a (0.06)

* Mean values of four replicates (*n* = 4); standard deviations are indicated in brackets. In each row, different letters (a, b) denote significant differences (*p* < 0.05). ** Abbreviations of sample names as expressed in Table 1. Other abbreviations: PUFAs (polyunsaturated FAs), STFAs (saturated FAs), MUFAs (monounsaturated FAs), and PI (polyene index).

**Table 4:** Primary and secondary lipid oxidation * in initial and canned fish, including different concentrations of cuttlefish ink (CI) in the packing medium **.

Initial or Canned Sample	Lipid Oxidation Index	
	Peroxide Value (Meq. Active Oxygen·kg^−1^ Lipids)	Thiobarbituric Acid Index (mg Malondialdehyde·kg^−1^ Muscle)
Initial fish	1.65 ab (0.34)	0.08 a (0.02)
CTR	1.10 a (0.18)	0.07 a (0.03)
CI-1	2.06 b (0.53)	0.08 a (0.03)
CI-2	1.37 ab (0.63)	0.09 a (0.03)
CI-3	1.53 ab (0.19)	0.10 a (0.02)
CI-4	1.98 ab (0.85)	0.13 a (0.03)

* Mean values of four replicates (*n* = 4); standard deviations are indicated in brackets. In each column, different letters (a, b) denote significant differences (*p* < 0.05). ** Abbreviations of sample names as expressed in Table 1.

**Table 5:** Colour changes * in initial and canned fish, including different concentrations of cuttlefish ink (CI) in the packing medium **.

Initial or Canned Sample	Colour Parameter		
	L*	a*	b*
Initial fish	46.35 a (0.96)	2.21 b (0.76)	−3.76 a (0.24)
CTR	81.40 c (2.03)	−1.07 a (0.51)	8.98 b (0.68)
CI-1	55.54 ab (8.39)	2.18 b (0.67)	6.81 b (1.79)
CI-2	63.48 b (6.09)	1.38 b (0.83)	7.31 b (1.06)
CI-3	62.46 b (4.58)	1.53 b (0.36)	8.66 b (1.95)
CI-4	60.39 b (5.50)	1.91 b (0.36)	7.39 b (0.99)

* Mean values of four replicates (*n* = 4); standard deviations are indicated in brackets. In each column, different letters (a, b, c) denote significant differences (*p* < 0.05). ** Abbreviations of sample names as expressed in Table 1.

## Data Availability

The original contributions presented in the study are included in the article, further inquiries can be directed to the corresponding author.
